# Hyperthyroidism is associated with breast cancer risk and mammographic and genetic risk predictors

**DOI:** 10.1186/s12916-020-01690-y

**Published:** 2020-08-25

**Authors:** Haomin Yang, Natalie Holowko, Felix Grassmann, Mikael Eriksson, Per Hall, Kamila Czene

**Affiliations:** 1 Department of Epidemiology and Health Statistics, School of Public Health, Fujian Medical University, Xuefu North Road 1, University Town, Fuzhou, 350122 China; 2 Department of Medical Epidemiology and Biostatistics, Karolinska Institutet, SE-17177 Stockholm, Sweden; 3 Department of Oncology, South General Hospital, SE-11883 Stockholm, Sweden

**Keywords:** Breast cancer, Hyperthyroidism, Mammographic density, Genetic pleiotropy

## Abstract

**Background:**

Despite the biological link between thyroid hormones and breast cancer cell proliferation shown in experimental studies, little is known about the association between hyperthyroidism and breast cancer, as well as its association with the most common mammographic and genetic risk predictors for breast cancer.

**Methods:**

This study estimates the incidence rate ratios (IRRs) of breast cancer among women diagnosed with hyperthyroidism, compared to those who are not, using two cohorts: a Swedish national cohort of the general female population (*n* = 3,793,492, 2002–2011) and the Karolinska Mammography Project for Risk Prediction of Breast Cancer (KARMA, *n* = 69,598, 2002–2017). We used logistic regression to estimate the odds ratios (ORs) of hyperthyroidism according to the mammographic and genetic risk predictors for breast cancer.

**Results:**

An increased risk of breast cancer was observed in patients in the national cohort with hyperthyroidism (IRR = 1.23, 95% CI = 1.12–1.36), particularly for toxic nodular goiter (IRR = 1.38, 95% CI = 1.16–1.63). Hyperthyroidism was associated with higher body mass index, early age at first birth, and lower breastfeeding duration. Higher mammographic density was observed in women with toxic nodular goiter, compared to women without hyperthyroidism. Additionally, among genotyped women without breast cancer in the KARMA cohort (*N* = 11,991), hyperthyroidism was associated with a high polygenic risk score (PRS) for breast cancer overall (OR = 1.98, 95% CI = 1.09–3.60) and for estrogen receptor-positive specific PRS (OR = 1.90, 95% CI = 1.04–3.43).

**Conclusion:**

Hyperthyroidism is associated with an increased risk of breast cancer, particularly for patients with toxic nodular goiter. The association could be explained by higher mammographic density among these women, as well as pleiotropic genetic variants determining shared hormonal/endocrine factors leading to the pathology of both diseases.

## Background

Breast cancer is the most common cancer diagnosed among women worldwide and the leading cause of cancer deaths among women [1]. Breast cancer is usually regarded as a hormone-related cancer, with approximately 70–80% of cases being estrogen receptor-positive [2]. Experimental studies show that thyroid hormones can stimulate cell proliferation in breast tissue [3, 4]. At high serum concentrations, thyroid hormones can have estrogen-like effects [5–7], which induce the expression of progesterone receptors [6] and can enhance estradiol-mediated effects on cell proliferation [3, 8].

Several population-based studies have reported hyperthyroidism (excessive production of thyroid hormones) to be associated with breast cancer [9–12], while others have found no association [13–15]. The disparity of findings may be explained by differences in study design, small sample sizes, or using a specific subgroup of women. Moreover, no study to date has examined the impact of different subtypes of hyperthyroidism on breast cancer risk, which may differ in their pathological process.

Although it is shown that thyroid hormones are associated with breast tissue proliferation and a subsequent increase in breast cancer risk [3, 4], the association between hyperthyroidism and mammographic features of breast tissue is less studied [16]. Mammographic density refers to the percentage of radiologically dense epithelium, stroma, and connective tissues identified in a mammogram. Mammographic density is a widely used mammographic feature for breast cancer risk prediction and is considered an intermediate phenotype for breast cancer [17, 18]. It can therefore be a powerful proxy when investigating the association between hyperthyroidism and breast cancer.

Besides the direct effect of thyroid hormones on breast cancer risk, the association between hyperthyroidism and breast cancer could also be explained by genetic pleiotropic pathways leading to both hyperthyroidism and breast cancer [19]. A recent study has shown an association between thyroid-related single nucleotide polymorphisms (SNPs) and breast cancer risk [20]. However, it is still unclear whether a genetic predisposition to breast cancer is associated with hyperthyroidism, which is important for examining a potential pleiotropic genetic effect.

In this study, we investigated mechanisms behind the association between hyperthyroidism and breast cancer. Specifically, whether a genetic susceptibility to breast cancer, measured using polygenic risk score and mammographic density, could explain some of this association, while being able to account for important factors such as menopausal status, body mass index (BMI), and reproductive health.

## Methods

### Study populations

To study the association between hyperthyroidism and breast cancer risk, we used two cohorts: (1) a Swedish national cohort of the general female population and (2) a mammographic screening-based cohort, Karolinska Mammography Project for Risk Prediction of Breast Cancer (KARMA) (Additional file 1: Fig. S1).

The national cohort included all women above age 20 years, who lived in Sweden between 2002 and 2011 (*N* = 3,793,492). Having a main diagnosis of hyperthyroidism was retrieved from the Swedish Patient Register and classified according to the International Classification of Diseases (ICD) code E05. Types of hyperthyroidism were defined by the ICD codes E050 (Graves’ disease), E051–E052 (toxic nodular goiter), and E053–E059 (other or unspecified types). Follow-up of the cohort ended on the date of breast cancer diagnosis, date of death, date of emigration, or end of follow-up (December 31, 2011), whichever came first. Information on breast cancer diagnosis, death, and emigration was obtained by using unique personal identification numbers to link the cohort to the Swedish Cancer Register, Swedish Cause of Death Register, and the Swedish Migration Register. The Swedish Cancer Register was founded in 1958. The completeness and accuracy of this register are estimated at 99 and 96%, respectively [21]. The ICD-7 code 170 was used to identify breast cancer diagnoses in the cancer register and to exclude breast cancer cases before 2002.

KARMA is a screening-based cohort, formed through an invitation to all women participating in a mammographic screening or clinical mammography in one of four hospitals in Sweden between January 2011 and March 2013 [22]. During the recruitment period, 70,877 women consented to join the study, with a participation rate of 34%. Aside from mammographic imaging and blood sample collection, participants also answered a web-based questionnaire including demographic, anthropometric, reproductive, lifestyle, and familial risk factors related to breast cancer. This cohort was linked to the Swedish Patient Register to retrieve diagnoses of hyperthyroidism. Breast cancer cases in the cohort were identified through linkage to the Swedish Cancer Register. For consistency with the national cohort, follow-up of the KARMA cohort also started from 2002, and ended with the same criteria as the national cohort, except for an extension of the follow-up time until Dec 29, 2017. We therefore excluded women with hyperthyroidism or breast cancer before 2002, ending with the final study population of 69,598 women. Characteristics of women in the national and the KARMA cohort are shown in Additional file 1: Table S1.

### Polygenic risk score

Blood samples from a subset of 11,991 women who did not have breast cancer when they joined the KARMA cohort were genotyped. These women were part of the Breast Cancer Association Consortium and were randomly selected as controls for the genome-wide association studies (GWAS) [23, 24]. Genotyping was performed using a custom Illumina iSelect array (iCOGS), comprising 211,155 SNPs [23], or OncoArray comprising of 499,170 SNPs [24]. Details of the array design, sample handling, quality control processes, and imputation of non-genotyped variants are described elsewhere [23, 25]. Hyperthyroidism cases were defined as women who were diagnosed with hyperthyroidism from 2002 to 2017, while the controls were women without a diagnosis of hyperthyroidism. To assess whether hyperthyroidism is associated with a genetic predisposition of breast cancer, we selected 171 genome-wide significant SNPs reported in a recent meta-analysis of breast cancer GWAS for constructing polygenic risk scores, for breast cancer overall and by estrogen receptor (ER) status [25]. For all individuals, a weighted polygenic risk score (PRS) was calculated using the following formula:

$$ \mathrm{PRS}={\beta}_1{x}_1+{\beta}_2{x}_2+.\dots {\beta}_k{x}_k+{\beta}_n{x}_n $$

where *β* is the per-allele log odds ratio (OR) of breast cancer-associated risk allele for SNP _*k*_, *x*_*k*_ is the number of alleles for the same SNP (0, 1, 2), and _*n*_ is the total number of the breast cancer SNPs included in the profile. The SNPs and corresponding betas used to derive the PRS are summarized in Additional file 1: Table S2. For the analyses, PRS was categorized into quartiles.

### Mammographic density

Full-field digital mammograms from mediolateral oblique views of the left and right breasts in the most recent screening before 2017 were used. For the KARMA cohort, we used the area-based STRATUS method to measure mammographic density [26]. Percent density was calculated by dividing the dense area by the total breast area in the mammogram and further categorized into quartiles. For this part of the study, we excluded women with any cancer diagnosis, as well as those who had a breast enlargement, reduction, or surgery, resulting in a final study population of 51,928. Only women diagnosed with hyperthyroidism before the latest screening were considered as cases.

The study was approved by the Regional Ethical Review Board in Stockholm, Sweden (Dnr 2010/958-31/1, 2012/217/-32/2, and 2014/1401-32).

### Statistical analysis

For both cohorts, the incidence rate ratio (IRR) of breast cancer among hyperthyroidism patients was calculated using Poisson regression, using attained age as the time scale and adjusting for calendar period (3-year categories). For these analyses, hyperthyroidism was treated as a time-varying exposure, in which the exposed person-time was counted from 3 months after the hyperthyroidism diagnosis (index date) and the unexposed person-time was counted from Jan 1, 2002, and ended on the date of breast cancer diagnosis, date of death, date of emigration, index date, or end of follow-up, whichever came first. The analysis was further stratified by menopausal status, age and time since hyperthyroidism diagnosis, and type of hyperthyroidism. In the analyses, Model 1 adjusted for calendar period and Model 2 further adjusted for BMI, age at menarche, number of births, family history of breast cancer, oral contraceptive use, hormone replacement treatment, and having had a benign breast disease.

The association between hyperthyroidism and various lifestyle risk factors for breast cancer were assessed among KARMA women using logistic regression models. These risk factors included having had a benign breast disease, number of births, age at first birth, breastfeeding duration, BMI, family history of breast cancer, oral contraceptive use, hormone replacement therapy use, age at menarche, and menopausal status. Two models were conducted for this analysis, a univariable model and a multivariable model adjusting for all these risk factors simultaneously.

Given that mammographic density is causally related to breast cancer risk, we also tested the association between having a prior diagnosis of hyperthyroidism and mammographic density among KARMA women using logistic regression models. Model 1 was the univariable model, Model 2 adjusted for age at mammogram and BMI, and Model 3 further adjusted for age at menarche, number of births, family history of breast cancer, oral contraceptive use, hormone replacement treatment, and having had a benign breast disease. Linear regression models were also used to test the effect of hyperthyroidism on mammographic density (square root transformed) as a continuous variable.

To test the association between hyperthyroidism and having a genetic predisposition to breast cancer, we used logistic regression models to calculate the ORs of hyperthyroidism among KARMA women who had been genotyped, by quartiles of PRS for breast cancer overall, for ER-positive cancers, and for ER-negative cancers. These associations were adjusted for age, the first three principle components, and for genotyping array. We further adjusted for age at menarche, number of births, family history of breast cancer, BMI, menopausal status, oral contraceptive use, hormone replacement treatment, and having had a benign breast disease.

Statistical analyses were performed using SAS (version 9.4; SAS Institute Inc., Cary, NC, USA) and Stata software (version 15.0; Stata Corporation, College Station, TX).

## Results

In the national cohort, 389 cases of breast cancer patients were observed during 133,154 person years following a diagnosis of hyperthyroidism, corresponding to an incidence rate of 2.9/1000 person years. Patients with hyperthyroidism experienced a 23% increased risk of breast cancer (IRR = 1.23, 95% CI = 1.12–1.36). In the KARMA cohort, there were 36 cases of breast cancer after hyperthyroidism diagnosis during a follow-up time of 7930 person years, corresponding to an incidence rate of 4.5/1000 person years. KARMA women with a diagnosis of hyperthyroidism also had a 22% increased risk of breast cancer, although the association was not statistically significant in the multivariable adjusted model (IRR = 1.22, 95% CI = 0.88–1.70). In both cohorts, women diagnosed with hyperthyroidism aged younger than 40 years had a significantly increased risk of breast cancer, while there was no strong difference for the risk of breast cancer according to menopausal status (Table 1). Analyses by type of hyperthyroidism in the national cohort indicated a stronger association between breast cancer and toxic nodular goiter (IRR = 1.38, 95% CI = 1.16–1.63).

**Table 1:** Risk of breast cancer in women in the national and KARMA cohorts with a diagnosis of hyperthyroidism, compared to women without hyperthyroidism

	Swedish national cohort *N* = 3,793,492 (2002–2011)			KARMA cohort *N* = 69,598 (2002–2017)			
	No. HT	No. BC	IRR (95% CI) Model 1	No. HT	No. BC	IRR (95% CI) Model 1	IRR (95% CI) Model 2
Overall	28,136	389	**1.23 (1.12–1.36)**	1034	36	1.23 (0.88–1.70)	1.22 (0.88–1.70)
Premenopausal	11,298	54	1.28 (0.98–1.68)	353	6	1.14 (0.51–2.54)	1.11 (0.50–2.48)
Postmenopausal	19,668	335	**1.23 (1.10–1.36)**	1010	30	1.27 (0.88–1.82)	1.26 (0.88–1.81)
By age (years)							
< 40	6659	25	**1.51 (1.02–2.24)**	106	6	**3.16 (1.41–7.05)**	**3.14 (1.41–7.02)**
40–55	7284	94	1.15 (0.94–1.41)	481	9	0.74 (0.38–1.42)	0.73 (0.38–1.40)
> 55	14,633	270	**1.24 (1.10–1.40)**	447	21	1.38 (0.90–2.12)	1.38 (0.90–2.13)
By years since hyperthyroidism diagnosis							
0–2	28,136	123	**1.23 (1.03–1.47)**	1034	10	1.70 (0.92–3.17)	1.69 (0.91–3.14)
2–5	22,648	157	**1.25 (1.07–1.46)**	915	9	1.02 (0.53–1.96)	1.01 (0.52–1.94)
>5	14,026	109	1.21 (1.00–1.46)	696	17	1.16 (0.72–1.87)	1.16 (0.72–1.87)
By types of hyperthyroidism							
Graves’ disease	16,552	201	**1.17 (1.02–1.35)**	736	27	1.34 (0.92–1.96)	1.33 (0.91–1.95)
Toxic nodular goiter	7113	130	**1.38 (1.16–1.63)**	278	13	1.43 (0.83–2.47)	1.43 (0.83–2.47)
Others or unspecified	14,592	187	**1.20 (1.04–1.38)**	377	7	0.78 (0.37–1.64)	0.78 (0.37–1.63)

Hyperthyroidism patients were identified by the main diagnosis given in the inpatient and outpatient registers. IRRs were calculated by comparing hyperthyroidism patients to women without hyperthyroidism using Poisson regression, using age as the underlying time scale. In the analyses stratified by menopausal status, women with age younger than 50 in the national cohort were considered as premenopausal women, while in the KARMA cohort, the exact age at menopause was used. Model 1 adjusted for calendar period, and Model 2 further adjusted for BMI, age at menarche, number of births, family history of breast cancer, hormone replacement therapy use, oral contraceptive use, and benign breast disease. Statistically significant results are bolded

*HT* hyperthyroidism, *BC* breast cancer, *IRR* incidence rate ratio, *CI* confidence interval

Among the major risk factors for breast cancer, a diagnosis of hyperthyroidism was significantly associated with obesity (BMI > 30, OR = 1.28, 95% CI = 1.01–1.61), which was predominantly driven by toxic nodular goiters (Additional file 1: Table S3). Other reproductive factors including early age at first birth, breastfeeding for less than a month, and menopausal status were also associated with hyperthyroidism (Table 2).

**Table 2:** The association between hyperthyroidism and major breast cancer risk predictors in KARMA cohort (*N* = 67,518)

Variable names	No. of non-hyperthyroidism	No. of hyperthyroidism	OR1 (95% CI)	OR2 (95% CI)
Benign breast disease				
No	47,345	451	1.00 (REF)	1.00 (REF)
Yes	13,820	135	1.03 (0.85–1.24)	1.01 (0.83–1.22)
Number of births				
0	7836	78	1.00 (REF)	1.00 (REF)
1	9032	96	1.07 (0.79–1.44)	1.07 (0.79–1.45)
2	29,471	296	1.01 (0.79–1.30)	1.03 (0.80–1.33)
> 2	15,245	130	0.86 (0.65–1.14)	0.86 (0.65–1.14)
Age at first birth*(years)				
< 25	18,167	206	1.00 (REF)	1.00 (REF)
25–29	19,023	161	**0.75 (0.61–0.92)**	**0.78 (0.63–0.97)**
≥ 30	16,536	155	0.83 (0.67–1.02)	0.90 (0.71–1.14)
Breastfeeding duration* (months)				
< 1	4557	60	1.00 (REF)	1.00 (REF)
1–6	19,276	210	0.83 (0.62–1.10)	0.86 (0.64–1.15)
> 6	29,220	249	**0.65 (0.49–0.86)**	**0.73 (0.54–0.98)**
BMI (kg/cm^2^) category				
< 18.5	639	6	1.03 (0.46–2.32)	1.03 (0.46–2.33)
18.5–25	34,076	310	1.00 (REF)	1.00 (REF)
25–30	19,518	188	1.06 (0.88–1.27)	1.03 (0.86–1.24)
> 30	7974	96	**1.32 (1.05–1.67)**	**1.28 (1.01–1.61)**
Family history of breast cancer				
No	51,994	495	1.00 (REF)	1.00 (REF)
Yes	7809	77	1.04 (0.81–1.32)	1.02 (0.80–1.30)
Oral contraceptive use				
No	12,162	128	1.00 (REF)	1.00 (REF)
Yes	49,271	471	0.91 (0.75–1.11)	0.95 (0.78–1.16)
Hormone replacement therapy use				
No	43,867	414	1.00 (REF)	1.00 (REF)
Yes	17,269	180	1.10 (0.93–1.32)	0.99 (0.82–1.20)
Age at menarche (years)				
< 12	20,998	217	1.00 (REF)	1.00 (REF)
13–16	36,565	345	0.91 (0.77–1.08)	0.92 (0.77–1.09)
> 16	2966	30	0.98 (0.67–1.44)	0.97 (0.66–1.43)
Menopausal status				
Pre-menopausal	27,283	225	1.00 (REF)	1.00 (REF)
Peri-menopausal	3753	40	1.29 (0.92–1.81)	1.29 (0.90–1.86)
Post-menopausal	35,830	387	**1.31 (1.11–1.54)**	**1.36 (1.02–1.81)**
History of irregular menstrual periods				
No	53,603	520	1.00 (REF)	1.00 (REF)
Yes	7113	75	1.09 (0.85–1.39)	1.11 (0.87–1.42)

OR1 was calculated using a univariate model while OR2 was calculated with a multivariate model including all risk predictors simultaneously. The analysis was restricted to KARMA women who had not had a breast cancer diagnosis when they entered the study

*OR* odds radio, *BMI* body mass index

*Analyses for age at first birth and breastfeeding duration were limited to parous women

When investigating the association between hyperthyroidism and mammographic density, no overall statistically significant association was observed (Table 3). However, a prior diagnosis of toxic nodular goiter was significantly associated with high mammographic density (OR for the highest quartile = 1.79, 95% CI = 1.03–3.11, *p* for continuous = 0.01), even after adjusting for all other potential risk factors for breast cancer (Additional file 1: Table S4).

**Table 3:** The association between hyperthyroidism and mammographic density among KARMA women without a cancer diagnosis (*n* = 51,928)

	No	Yes	OR (95% CI)		
			Model 1	Model 2	Model 3
**Mammographic density**					
**Q1**	12,797	186	1.00 (REF)	1.00 (REF)	1.00 (REF)
**Q2**	12,801	182	0.98 (0.80–1.20)	1.10 (0.89–1.36)	1.09 (0.88–1.35)
**Q3**	12,807	174	0.94 (0.76–1.15)	1.17 (0.93–1.48)	1.16 (0.92–1.47)
**Q4**	12,825	156	0.84 (0.68–1.04)	1.15 (0.88–1.51)	1.14 (0.87–1.49)
***P*for square root continuous**			0.13	0.13	0.20

Model 1 is the univariate model. Model 2 adjusted for age and BMI. Model 3 further adjusted for age at menarche, number of births, family history of breast cancer, hormone replacement therapy use, oral contraceptive use, and benign breast disease

Among KARMA women with genotyped data, a higher PRS for breast cancer was associated with hyperthyroidism (OR for the highest quartile = 1.98, 95% CI = 1.09–3.60). This association was mainly driven by the association between hyperthyroidism and ER-positive breast cancer (OR for the highest quartile = 1.90, 95% CI = 1.04–3.43) (Table 4). There was no association between hyperthyroidism and PRS for ER-negative breast cancer.

**Table 4:** The association between hyperthyroidism and breast cancer polygenic risk scores (PRS) among women in the KARMA cohort (*N* = 11,991)

	No.	No. case	OR1 (95% CI)	OR2 (95% CI)
PRS for BC overall				
Q1	2952	46	1.00 (REF)	1.00 (REF)
Q2	2952	46	1.05 (0.69–1.59)	1.08 (0.71–1.64)
Q3	2964	34	0.93 (0.55–1.56)	0.95 (0.56–1.60)
Q4	2937	60	**1.90 (1.05–3.45)**	**1.98 (1.09–3.60)**
*P* for trend			0.07	0.06
Standardized continuous			1.19 (0.96–1.48)	1.21 (0.97–1.50)
PRS for ER + BC				
Q1	2953	44	1.00 (REF)	1.00 (REF)
Q2	2948	50	1.19 (0.79–1.80)	1.22 (0.81–1.85)
Q3	2965	33	0.91 (0.54–1.53)	0.93 (0.55–1.57)
Q4	2939	59	**1.83 (1.01–3.30)**	**1.90 (1.04–3.43)**
*P* for trend			0.09	0.07
Standardized continuous			1.18 (0.96–1.46)	1.20 (0.97–1.48)
PRS for ER − BC				
Q1	2952	45	1.00 (REF)	1.00 (REF)
Q2	2958	41	0.92 (0.60–1.41)	0.93 (0.61–1.43)
Q3	2947	51	1.16 (0.77–1.75)	1.19 (0.79–1.80)
Q4	2948	49	1.12 (0.73–1.72)	1.15 (0.75–1.77)
*P* for trend			0.41	0.33
Standardized continuous			1.07 (0.92–1.25)	1.09 (0.93–1.27)

Analysis was performed among KARMA women without breast cancer and who had genotyped data. OR1s were adjusted for age, array of genotyping, and principle components. OR2s were additionally adjusted for BMI, menopausal status, age at menarche, number of births, family history of breast cancer, HRT use, oral contraceptive use, and benign breast disease. Statistically significant results are bolded

*No. case* the number of hyperthyroidism patients, *No.* the number of women without hyperthyroidism, *OR* odds ratio, *CI* confidence interval, *ER* estrogen receptor

## Discussion

Women diagnosed with hyperthyroidism had an increased risk of breast cancer, compared to the general population. This finding was more pronounced among those diagnosed at a younger age and with a toxic nodular goiter. Hyperthyroidism was also associated with breast cancer risk predictors such as BMI, age at first birth, and duration of breastfeeding. The association between mammographic density and hyperthyroidism was only observed in women with a diagnosis of toxic nodular goiter. Additionally, having hyperthyroidism was associated with a high PRS for breast cancer, particularly for ER-positive breast cancer.

Our estimates for an increased risk of breast cancer among women diagnosed with hyperthyroidism were the same when analyzing data from the national and KARMA cohorts, which is also consistent with previous estimates in Denmark and Taiwan [10, 12]. This association, however, was not statistically significant in the KARMA cohort, probably due to a limited number of breast cancer patients (*N* = 36). The association between hyperthyroidism and breast cancer was further supported by an increased risk of breast cancer among women with high serum thyroxine (T4) [27, 28]. Despite this, the effect of serum triiodothyronine (T3) on breast cancer risk is still conflicting in different studies [29, 30], which might be influenced by non-thyroidal illness syndrome in cancer patients [31].

Several biological pathways may exist between hyperthyroidism and breast carcinogenesis. T3 could activate the PI3K pathways mediated by integrin αvβ3 and stimulate breast cancer cell invasion [32]. In addition, T4 induces MAPK-mediated nuclear ERα phosphorylation and promotes cell proliferation to an extent comparable to the effect of estradiol [33], which could be inhibited by the T4 analog tetrac [34].

In addition to the known association between thyroid hormones and breast cancer risk, it has been hypothesized that I-131 (radioactive iodine) treatment for hyperthyroidism may also increase the risk of breast cancer. However, majority of the studies did not find a significant association between I-131 treatment and breast cancer [35–40]. After surgery or radioactive iodine treatment, hyperthyroidism patients may subsequently reach a hypothyroid state and thus be prescribed thyroid replacement medication (e.g., levothyroxine) [41]. Despite this, a recent meta-analysis did not find an increased risk of breast cancer after subsequent hypothyroidism [42], nor following treatment with levothyroxine [43]. Moreover, considering the potential late effect of treatment on breast cancer, the observed association between hyperthyroidism and breast cancer in our study (particularly for short term risk) cannot be explained by the effect of treatment.

In this study, hyperthyroidism was associated with several risk factors for breast cancer. Both Graves’ disease and toxic nodular goiter are strongly influenced by pregnancy [44, 45], which could result in early cessation of breastfeeding and supports our finding of an association between reduced breastfeeding duration and hyperthyroidism. The strong association between hyperthyroidism and breast cancer observed among women aged below 40 years, in both the national and KARMA cohorts, further suggests that closer surveillance for breast cancer may be useful among women diagnosed with hyperthyroidism during their reproductive years.

We found a significantly higher BMI among patients with hyperthyroidism, despite previous knowledge that reduced weight is associated with hyperthyroidism [46]. This disparity could be explained by treatments for hyperthyroidism, which might eventually result in excess weight gain for patients [47, 48]. Moreover, the association between hyperthyroidism and BMI was mainly driven by toxic nodular goiters. This finding is consistent with previous studies indicating that obesity is associated with thyroid nodules [49, 50].

Independent of BMI, toxic nodular goiters were associated with higher mammographic density. While one previous study did not find a significant association between thyroid disorders (including hyperthyroidism and thyroid nodules) and mammographic density, this is likely due to having a limited sample size [16]. This finding in the KARMA cohort was consistent with the pronounced association between breast cancer and toxic nodular goiter in the national cohort. Unlike Graves’ disease which is an autoimmune disease, toxic nodular goiters are likely to be hormone-related, and therefore more closely linked to breast cancer. Nodular thyroid diseases are associated with benign breast disease and breast cancer [51, 52], and some shared pathways are involved in the proliferation of thyroid and breast tissues [4, 53, 54]. Hence, the association between hyperthyroidism and breast cancer could result from both a hormonal effect and tissue proliferation and, therefore, possibly mediated through mammographic density.

Additionally, we found a significant association between breast cancer PRS and hyperthyroidism. Among those GWAS significant SNPs for breast cancer, previous studies show that one SNP in the ABO gene is associated with thyroid function [55, 56]. Another gene overlapping the GWAS for thyroid-stimulating hormone and breast cancer is IGFBP5 [25, 56], indicating the shared growth hormone/insulin-like growth factor (GH/IGF) pathways between thyroid function and breast cancer. Although insignificant at the GWAS level, one SNP in the DIO1 gene has been associated with both free T4 and breast cancer [20], suggesting the role of deiodinase activity in the association between thyroid function and breast cancer [57]. Overall, given the genetic association was only significant for the ER-positive breast cancer PRS, and not for ER-negative PRS, we hypothesize that genetic pleiotropy between hyperthyroidism and breast cancer is involved in the shared hormonal/endocrine pathways for both diseases.

Considering the phenotypic and genetic associations between hyperthyroidism and breast cancer, as well as the beneficial effect of introduced euthyroid hypothyroxinemia in the setting of breast cancer [58], hyperthyroidism should be considered in the evaluation of women’s risk of breast cancer. Despite concerns of possible over-diagnosis and anxiety caused by mammographic screening [59, 60], better adherence to the scheduled breast cancer screening program is recommended for patients with hyperthyroidism, in order to detect breast cancer at an early stage.

The main strength of our study is having a large sample size and a population-based cohort design, including both Swedish health registers and a mammographic screening cohort. Other strengths include the KARMA cohort containing rich lifestyle, mammographic and genetic data, which allowed us to explore underlying mechanisms for the associations investigated. We also acknowledge several limitations. The validity of ICD codes in the Swedish patient register is about 85–95% [61], which indicates potential misclassification. Therefore, we tried to minimize any potential misclassification by using main diagnoses only. Due to increased medical surveillance of patients with hyperthyroidism, these women may be diagnosed earlier with breast cancer than women without hyperthyroidism. While this possible bias might slightly shift the temporal risk pattern among women with hyperthyroidism, it would not strongly influence the overall association. An older age at breast cancer diagnosis among patients with hyperthyroidism (see Additional file 1: Table S1) further argues against this surveillance bias. In the national cohort, we were not able to adjust for breast cancer risk factors. Nevertheless, in the KARMA cohort which contains detailed information on these confounders, multivariable adjustment did not change the point estimates, indicating a weak confounding effect of these breast cancer risk factors. In the analysis of breast cancer risk using the KARMA cohort, we started the follow-up before women entered the KARMA study, which could have introduced survivor bias. However, we believe the effect of any such bias would be minimal, given that hyperthyroidism is not a deadly disease and the same estimates were found using the national cohort.

## Conclusion

In conclusion, we found that women diagnosed with hyperthyroidism were associated with an increased risk of breast cancer and major predictors for breast cancer, including mammographic density and polygenic risk score. These findings may partly be explained by shared genetic and hormonal factors between these two diseases and may contribute to a more comprehensive understanding of hormonal/endocrinal factors contributing to breast cancer risk.

## Supplementary information

**Additional file 1: Table S1.** Characteristics of women in the national and KARMA cohorts, by hyperthyroidism status. **Table S2.** Single nucleotide polymorphisms (SNPs) used for constructing the polygenic risk score (PRS) for breast cancer. **Table S3.** The association between hyperthyroidism and major breast cancer risk predictors among the KARMA cohort, by type of hyperthyroidism (*N* = 67,518). **Table S4.** The association between hyperthyroidism and mammographic density among KARMA women without cancer, by type of hyperthyroidism (*n* = 51,928). **Figure S1.** Sample attrition for the analyses using the KARMA cohort

## Data Availability

The KARMA data used in the present study are available from the corresponding author upon reasonable request. The register-based nationwide cohort datasets linked and analyzed in the current study are not publicly available due to Swedish law but are available by applying through the Swedish National Board of Health and Welfare and Statistics Sweden. Detailed information on data application can be found using the following links https://bestalladata.socialstyrelsen.se/data-for-forskning/ and https://www.scb.se/vara-tjanster/bestalla-mikrodata/.

## Abbreviations

BMI: Body mass index
CI: Confidence interval
ER: Estrogen receptor
GWAS: Genome-wide association studies
ICD: International Classification of Diseases
IRR: Incidence rate ratio
KARMA: Karolinska Mammography Project for Risk Prediction of Breast Cancer
OR: Odds ratio
PRS: Polygenic risk score
SNPs: Single nucleotide polymorphisms
T3: Triiodothyronine
T4: Thyroxine

## References

[1] Global Burden of Disease Collaboration: Global, regional, and national cancer incidence, mortality, years of life lost, years lived with disability, and disability-adjusted life-years for 29 cancer groups, 1990 to 2017: a systematic analysis for the global burden of disease study. JAMA Oncology 2019;5(12):1749–68. 10.1001/jamaoncol.2019.2996 PMC6777271 31560378

[2] Allred DC, Brown P, Medina D (2004). The origins of estrogen receptor alpha-positive and estrogen receptor alpha-negative human breast cancer. Breast Cancer Res.

[3] Hall LC, Salazar EP, Kane SR, Liu N (2008). Effects of thyroid hormones on human breast cancer cell proliferation. J Steroid Biochem Mol Biol.

[4] Conde I, Paniagua R, Zamora J, Blanquez MJ, Fraile B, Ruiz A, Arenas MI (2006). Influence of thyroid hormone receptors on breast cancer cell proliferation. Ann Oncol.

[5] Dinda S, Sanchez A, Moudgil V (2002). Estrogen-like effects of thyroid hormone on the regulation of tumor suppressor proteins, p53 and retinoblastoma, in breast cancer cells. Oncogene.

[6] Nogueira CR, Brentani MM (1996). Triiodothyronine mimics the effects of estrogen in breast cancer cell lines. J Steroid Biochem Mol Biol.

[7] Ditsch N, Liebhardt S, Von Koch F, Lenhard M, Vogeser M, Spitzweg C, Gallwas J, Toth B (2010). Thyroid function in breast cancer patients. Anticancer Res.

[8] Shao ZM, Sheikh MS, Rishi AK, Dawson MI, Li XS, Wilber JF, Feng P, Fontana JA (1995). Thyroid hormone enhancement of estradiol stimulation of breast carcinoma proliferation. Exp Cell Res.

[9] Journy NMY, Bernier MO, Doody MM, Alexander BH, Linet MS, Kitahara CM (2017). Hyperthyroidism, hypothyroidism, and cause-specific mortality in a large cohort of women. Thyroid.

[10] Sogaard M, Farkas DK, Ehrenstein V, Jorgensen JO, Dekkers OM, Sorensen HT (2016). Hypothyroidism and hyperthyroidism and breast cancer risk: a nationwide cohort study. Eur J Endocrinol.

[11] Kim EY, Chang Y, Lee KH, Yun JS, Park YL, Park CH, Ahn J, Shin H, Ryu S. Serum concentration of thyroid hormones in abnormal and euthyroid ranges and breast cancer risk: a cohort study. Int J Cancer. 2019;145(12):3257–66. 10.1002/ijc.32283 30882890

[12] Weng C-H, Chen Y-H, Lin C-H, Luo X, Lin T-H (2018). Thyroid disorders and breast cancer risk in Asian population: a nationwide population-based case–control study in Taiwan. BMJ Open.

[13] Simon MS, Tang M-TC, Bernstein L, Norman SA, Weiss L, Burkman RT, Daling JR, Deapen D, Folger SG, Malone K *et al*: Do thyroid disorders increase the risk of breast cancer? *Cancer Epidemiology Biomarkers &amp;* Prevention 2002, 11(12):1574–1578. 12496046

[14] Hellevik AI, Åsvold BO, Bjøro T, Romundstad PR, Nilsen TIL, Vatten LJ: Thyroid function and cancer risk: a prospective population study. *Cancer Epidemiology Biomarkers &amp;* Prevention 2009, 18(2):570–574. 10.1158/1055-9965.EPI-08-0911 19155436

[15] Fang Y, Yao L, Sun J, Yang R, Chen Y, Tian J, Yang K, Tian L (2017). Does thyroid dysfunction increase the risk of breast cancer? A systematic review and meta-analysis. J Endocrinol Investig.

[16] Pedraza-Flechas AM, Lope V, Vidal C, Sánchez-Contador C, Santamariña C, Pedraz-Pingarrón C, Moreo P, Ascunce N, Miranda-García J, Llobet R (2017). Thyroid disorders and mammographic density in Spanish women: Var-DDM study. Breast.

[17] Tamimi RM, Byrne C, Colditz GA, Hankinson SE (2007). Endogenous hormone levels, mammographic density, and subsequent risk of breast cancer in postmenopausal women. J National Cancer Institute.

[18] Rice MS, Bertrand KA, VanderWeele TJ, Rosner BA, Liao X, Adami HO, Tamimi RM (2016). Mammographic density and breast cancer risk: a mediation analysis. Breast Cancer Res.

[19] Chaker L, Visser TJ (2016). Thyroid dysfunction and breast cancer risk — an unfinished story. Nat Rev Endocrinol.

[20] Brandt J, Borgquist S, Almgren P, Forsti A, Huss L, Melander O, Manjer J (2018). Thyroid-associated genetic polymorphisms in relation to breast cancer risk in the Malmo Diet and Cancer Study. Int J Cancer.

[21] Garne JP, Aspegren K, Moller T (1995). Validity of breast-cancer registration from one hospital into the Swedish National Cancer Registry 1971-1991. Acta Oncol.

[22] Gabrielson M, Eriksson M, Hammarstrom M, Borgquist S, Leifland K, Czene K, Hall P. Cohort profile: the Karolinska Mammography Project for Risk Prediction of Breast Cancer (KARMA). Int J Epidemiol. 2017;46(6):1740–41g. 10.1093/ije/dyw357 PMC5837703 28180256

[23] Michailidou K, Hall P, Gonzalez-Neira A, Ghoussaini M, Dennis J, Milne RL, Schmidt MK, Chang-Claude J, Bojesen SE, Bolla MK (2013). Large-scale genotyping identifies 41 new loci associated with breast cancer risk. Nat Genet.

[24] Amos CI, Dennis J, Wang Z, Byun J, Schumacher FR, Gayther SA, Casey G, Hunter DJ, Sellers TA, Gruber SB (2017). The OncoArray Consortium: a network for understanding the genetic architecture of common cancers. Cancer Epidemiol Biomarkers Prevention.

[25] Michailidou K, Lindstrom S, Dennis J, Beesley J, Hui S, Kar S, Lemacon A, Soucy P, Glubb D, Rostamianfar A (2017). Association analysis identifies 65 new breast cancer risk loci. Nature.

[26] Eriksson M, Czene K, Pawitan Y, Leifland K, Darabi H, Hall P (2017). A clinical model for identifying the short-term risk of breast cancer. Breast Cancer Research.

[27] Tosovic A, Becker C, Bondeson A-G, Bondeson L, Ericsson U-B, Malm J, Manjer J (2012). Prospectively measured thyroid hormones and thyroid peroxidase antibodies in relation to breast cancer risk. Int J Cancer.

[28] Khan SR, Chaker L, Ruiter R, Aerts JGJV, Hofman A, Dehghan A, Franco OH, Stricker BHC, Peeters RP (2016). Thyroid function and cancer risk: the Rotterdam study. J Clin Endocrinol Metabolism.

[29] Tosovic A, Bondeson A-G, Bondeson L, Ericsson U-B, Malm J, Manjer J (2010). Prospectively measured triiodothyronine levels are positively associated with breast cancer risk in postmenopausal women. Breast Cancer Res.

[30] Ortega-Olvera C, Ulloa-Aguirre A, Angeles-Llerenas A, Mainero-Ratchelous FE, Gonzalez-Acevedo CE, Hernandez-Blanco ML, Ziv E, Aviles-Santa L, Perez-Rodriguez E, Torres-Mejia G (2018). Thyroid hormones and breast cancer association according to menopausal status and body mass index. Breast Cancer Res.

[31] Hercbergs A, Mousa SA, Davis PJ (2018). Nonthyroidal illness syndrome and thyroid hormone actions at integrin αvβ3. J Clin Endocrinol Metabolism.

[32] Flamini MI, Uzair ID, Pennacchio GE, Neira FJ, Mondaca JM, Cuello-Carrión FD, Jahn GA, Simoncini T, Sanchez AM (2017). Thyroid hormone controls breast cancer cell movement via integrin αV/β3/SRC/FAK/PI3-kinases. Hormones Cancer.

[33] Tang H-Y, Lin H-Y, Zhang S, Davis FB, Davis PJ (2004). Thyroid hormone causes mitogen-activated protein kinase-dependent phosphorylation of the nuclear estrogen receptor. Endocrinology.

[34] Bharali DJ, Yalcin M, Davis PJ, Mousa SA (2013). Tetraiodothyroacetic acid-conjugated PLGA nanoparticles: a nanomedicine approach to treat drug-resistant breast cancer. Nanomedicine (Lond).

[35] Lin C-Y, Lin C-L, Huang W-S, Kao C-H (2016). Risk of breast cancer in patients with thyroid cancer receiving or not receiving 131I treatment: a nationwide population-based cohort study. J Nuclear Med.

[36] Holm L-E, Hall P, Wiklund K, Lundell G, Berg G, Bjelkengren G, Cederquist E, Ericsson U-B, Hallquist A, Larsson L-G (1991). Cancer risk after iodine-131 therapy for hyperthyroidism. J National Cancer Institute.

[37] Ron E, Doody MM, Becker DV, Brill AB, Curtis RE, Goldman MB, Harris BS, Hoffman DA, McConahey WM, Maxon HR (1998). Cancer mortality following treatment for adult hyperthyroidism. Cooperative thyrotoxicosis therapy follow-up study group. JAMA.

[38] Kitahara CM, Berrington de Gonzalez A, Bouville A, Brill AB, Doody MM, Melo DR, Simon SL, Sosa JA, Tulchinsky M, Villoing D (2019). Association of radioactive iodine treatment with cancer mortality in patients with hyperthyroidism. JAMA Intern Med.

[39] Hall P, Berg G, Bjelkengren G, Boice JD, Ericsson UB, Hallquist A, Lidberg M, Lundell G, Tennvall J, Wiklund K (1992). Cancer mortality after iodine-131 therapy for hyperthyroidism. Int J Cancer.

[40] Gronich N, Lavi I, Rennert G, Saliba W (2020). Cancer risk after radioactive iodine treatment for hyperthyroidism: a cohort study. Thyroid.

[41] Abraham P, Avenell A, Park CM, Watson WA, Bevan JS (2005). A systematic review of drug therapy for Graves’ hyperthyroidism. Eur J Endocrinol.

[42] Thi-Van-Trinh T, Cari MK, Florent de V, Marie-Christine B-R, Neige J: Thyroid dysfunction and cancer incidence: a systematic review and meta-analysis. Endocr Relat Cancer 2020, 27(4):245–259. 10.1530/ERC-19-0417 32045361

[43] Weng C-H, Okawa ER, Roberts MB, Park SK, Umbricht CB, Manson JE, Eaton CB (2020). Breast cancer risk in postmenopausal women with medical history of thyroid disorder in the Women’s Health Initiative. Thyroid.

[44] Moleti M, Di Mauro M, Sturniolo G, Russo M, Vermiglio F (2019). Hyperthyroidism in the pregnant woman: maternal and fetal aspects. J Clin Translational Endocrinol.

[45] Kung AWC, Chau MT, Lao TT, Tam SCF, Low LCK (2002). The effect of pregnancy on thyroid nodule formation. J Clin Endocrinol Metabolism.

[46] De Leo S, Lee SY, Braverman LE (2016). Hyperthyroidism. Lancet.

[47] Torlinska B, Nichols L, Mohammed MA, McCabe C, Boelaert K (2019). Patients treated for hyperthyroidism are at increased risk of becoming obese: findings from a large prospective secondary care cohort. Thyroid.

[48] Dale J, Daykin J, Holder R, Sheppard MC, Franklyn JA (2001). Weight gain following treatment of hyperthyroidism. Clin Endocrinol.

[49] Kim JY, Jung EJ, Park ST, Jeong SH, Jeong CY, Ju YT, Lee YJ, Hong SC, Choi SK, Ha WS (2012). Body size and thyroid nodules in healthy Korean population. J Korean Surgical Society.

[50] Xu W, Chen Z, Li N, Liu H, Huo L, Huang Y, Jin X, Deng J, Zhu S, Zhang S (2015). Relationship of anthropometric measurements to thyroid nodules in a Chinese population. BMJ Open.

[51] Shi Y, Li X, Ran L, Arshad B, Li H, Xu Z, Zhao C, Wu Y, Wu H, Chen H (2017). Study on the status of thyroid function and thyroid nodules in chinese breast cancer patients. Oncotarget.

[52] Anil C, Guney T, Gursoy A (2015). The prevalence of benign breast diseases in patients with nodular goiter and Hashimoto’s thyroiditis. J Endocrinol Investig.

[53] Beristain AG, Molyneux SD, Joshi PA, Pomroy NC, Di Grappa MA, Chang MC, Kirschner LS, Prive GG, Pujana MA, Khokha R (2015). PKA signaling drives mammary tumorigenesis through Src. Oncogene.

[54] Ferrero S, Vaira V, Del Gobbo A, Vicentini L, Bosari S, Beck-Peccoz P, Mantovani G, Spada A, Lania AG (2015). Different expression of protein kinase A (PKA) regulatory subunits in normal and neoplastic thyroid tissues. Histol Histopathol.

[55] Zhao SX, Xue LQ, Liu W, Gu ZH, Pan CM, Yang SY, Zhan M, Wang HN, Liang J, Gao GQ (2013). Robust evidence for five new Graves’ disease risk loci from a staged genome-wide association analysis. Hum Mol Genet.

[56] Teumer A, Chaker L, Groeneweg S, Li Y, Di Munno C, Barbieri C, Schultheiss UT, Traglia M, Ahluwalia TS, Akiyama M (2018). Genome-wide analyses identify a role for SLC17A4 and AADAT in thyroid hormone regulation. Nat Commun.

[57] Debski MG, Pachucki J, Ambroziak M, Olszewski W, Bar-Andziak E (2007). Human breast cancer tissue expresses high level of type 1 5'-deiodinase. Thyroid.

[58] Hercbergs A, Mousa SA, Leinung M, Lin H-Y, Davis PJ (2018). Thyroid hormone in the clinic and breast cancer. Hormones Cancer.

[59] Tosteson ANA, Fryback DG, Hammond CS, Hanna LG, Grove MR, Brown M, Wang Q, Lindfors K, Pisano ED (2014). Consequences of false-positive screening mammograms. JAMA Intern Med.

[60] Løberg M, Lousdal ML, Bretthauer M, Kalager M (2015). Benefits and harms of mammography screening. Breast Cancer Res.

[61] Ludvigsson JF, Andersson E, Ekbom A, Feychting M, Kim JL, Reuterwall C, Heurgren M, Olausson PO (2011). External review and validation of the Swedish national inpatient register. BMC Public Health.

