# Supplementary material for: Hyperthyroidism is associated with breast cancer risk and mammographic and genetic risk predictors
Source: BMC Med. 2020 Aug 25;18:225. doi: 10.1186/s12916-020-01690-y (PMC7446157; doi:10.1186/s12916-020-01690-y)
Supplement: Supplementary file 1 — Additional file 1: Table S1. Characteristics of women in the national and KARMA cohorts, by hyperthyroidism status. Table S2. Single nucleotide polymorphisms (SNPs) used for constructing the polygenic risk score (PRS) for breast cancer. Table S3. The association between hyperthyroidism and major breast cancer risk predictors among the KARMA cohort, by type of hyperthyroidism (N = 67,518). Table S4. The association between hyperthyroidism and mammographic density among KARMA women without cancer, by type of hyperthyroidism (n = 51,928). Figure S1. Sample attrition for the analyses using the KARMA cohort [file 12916_2020_1690_MOESM1_ESM.docx]

**Additional file 1:**

**Table S1.** Characteristics of women in the national and KARMA cohorts, by hyperthyroidism status.

**Table S2.** Single nucleotide polymorphisms (SNPs) used for constructing the polygenic risk score (PRS) for breast cancer.

**Table S3.** The association between hyperthyroidism and major breast cancer risk predictors among the KARMA cohort, by type of hyperthyroidism (N=67,518)

**Table S4.** The association between hyperthyroidism and mammographic density among KARMA women without cancer, by type of hyperthyroidism (n=51,928)

**Fig. S1.** Sample attrition for analyses using the KARMA cohort

Table S1. Characteristics of women in the national and KARMA cohorts, by hyperthyroidism status.

|  | No. of women (%) | |
| --- | --- | --- |
| **Variable names** | Women without hyperthyroidism | hyperthyroidism patients |
| **National cohort** |  |  |
| Number of women | 3,765,356 | 28136 |
| Mean age at start of follow-up (SD) | 47.1 (20.4) | 55.5 (18.2) |
| Duration of follow-up (years) |  |  |
| Median (IQR) | 10.0 (0.9) | 4.6 (5.3) |
| Total no. of person years at risk | 32,757,108 | 133,154 |
| Cases of breast cancer | 62,232 | 389 |
| Mean Age at breast cancer diagnosis (SD) | 63.9 (13.9) | 65.1 (13.6) |
| **KARMA cohort** |  |  |
| Number of women | 68564 | 1034 |
| Mean age at start of follow-up (SD) | 44.6 (10.0) | 53.4 (10.7) |
| Duration of follow-up (years) |  |  |
| Median (IQR) | 16 (0) | 7.3 (8.3) |
| Total no. of person years at risk | 1,078,402 | 7930 |
| Cases of breast cancer | 3,426 | 36 |
| Mean Age at breast cancer diagnosis (SD) | 58.6 (10.2) | 59.1 (9.8) |

Abbreviations: SD = standard deviation; IQR = interquartile.

Table S2. Single nucleotide polymorphisms (SNPs) used for constructing the polygenic risk score (PRS) for breast cancer.

| SNP | Effect | Effect size | | |
| --- | --- | --- | --- | --- |
|  | Allele | Breast cancer overall | ER positive breast cancer | ER negative breast cancer |
| rs116095464 | C | 0.0592 | 0.06672 | 0.02797 |
| rs6597981 | G | 0.04502 | 0.04485 | 0.05631 |
| rs10069690 | T | 0.05673 | 0.02217 | 0.16997 |
| rs3215401 | AG | -0.07005 | -0.04898 | -0.12542 |
| rs11242675 | T | -0.00038 | -0.00015 | 0.01112 |
| rs3817198 | C | 0.04781 | 0.05229 | 0.02235 |
| rs6762644 | G | 0.05255 | 0.05571 | 0.04218 |
| rs2380205 | T | -0.02293 | -0.02244 | -0.02814 |
| rs16991615 | A | 0.09571 | 0.09456 | 0.09719 |
| rs67958007 | T | 0.08237 | 0.08479 | 0.08308 |
| rs113577745 | G | 0.07548 | 0.07238 | 0.06436 |
| rs9348512 | A | 0.00445 | 0.00437 | 0.01133 |
| rs616488 | G | -0.05785 | -0.04373 | -0.11584 |
| rs78269692 | C | 0.08354 | 0.08447 | 0.08618 |
| rs204247 | A | -0.03624 | -0.04246 | -0.00022 |
| rs2594714 | A | -0.02959 | -0.0255 | 0.00204 |
| rs12422552 | C | 0.05611 | 0.0472 | 0.04326 |
| rs13162653 | T | -0.01211 | -0.01128 | -0.02042 |
| rs3819405 | T | -0.04546 | -0.05277 | -0.03534 |
| rs2823093 | A | -0.06228 | -0.08213 | -4.00E-05 |
| rs67397200 | G | 0.02821 | -0.00863 | 0.15918 |
| rs4808801 | G | -0.07028 | -0.07817 | -0.04055 |
| rs2992756 | C | -0.06077 | -0.06729 | -0.03267 |
| rs12710696 | C | -0.02531 | -0.01406 | -0.03767 |
| rs2965183 | A | 0.04159 | 0.03758 | 0.05391 |
| rs2223621 | C | -0.03649 | -0.03317 | -0.01781 |
| rs7971 | G | -0.04136 | -0.04187 | -0.04227 |
| rs7072776 | G | -0.05064 | -0.06433 | 0.01509 |
| rs1011970 | T | 0.06341 | 0.06186 | 0.04527 |
| rs11814448 | C | 0.11579 | 0.13586 | 0.04141 |
| rs527616 | G | 0.03382 | 0.04652 | -0.00066 |
| rs1436904 | G | -0.04891 | -0.06154 | -0.00622 |
| rs6725517 | G | -0.04105 | -0.03481 | -0.06936 |
| rs71557345 | A | -0.08273 | -0.0891 | -0.09985 |
| rs4973768 | T | 0.10012 | 0.11215 | 0.04002 |
| rs7297051 | T | -0.11548 | -0.11092 | -0.13771 |
| rs17156577 | C | 0.04968 | 0.04987 | 0.04363 |
| rs9257408 | C | 0.01764 | 0.0215 | 0.03126 |
| rs4577244 | T | 0.01269 | 0.04425 | -0.07742 |
| rs17879961 | G | 0.22752 | 0.33284 | -0.14537 |
| rs146699004 | G | -0.03277 | -0.0363 | -0.04933 |
| rs9693444 | C | -0.06128 | -0.06986 | -0.01849 |
| rs132390 | T | -0.04099 | -0.03529 | -0.00593 |
| rs117618124 | C | -0.12186 | -0.11266 | -0.17106 |
| rs12493607 | C | 0.04756 | 0.06011 | -0.00459 |
| rs2012709 | T | 0.02069 | 0.03066 | -0.02316 |
| rs2284378 | C | 0.00256 | 0.00152 | 0.01317 |
| rs11571833 | T | 0.29858 | 0.24695 | 0.45564 |
| rs13365225 | G | -0.09442 | -0.09023 | -0.10422 |
| rs2236007 | A | -0.06871 | -0.06931 | -0.04949 |
| rs738321 | G | -0.05154 | -0.06933 | -0.00821 |
| rs6815814 | C | 0.05672 | 0.05045 | 0.05681 |
| chr22:39359355 | D10 | 0.09697 | 0.10288 | 0.09783 |
| rs72826962 | T | 0.18359 | 0.1859 | 0.12427 |
| rs6001930 | C | 0.1174 | 0.10799 | 0.12747 |
| rs4233486 | T | 0.03493 | 0.02991 | 0.03195 |
| rs73161324 | T | 0.054 | 0.03794 | 0.10099 |
| rs79724016 | G | -0.07592 | -0.09378 | -0.02294 |
| rs6507583 | G | -0.08015 | -0.10595 | -0.03273 |
| rs2532263 | A | -0.05417 | -0.05071 | -0.05071 |
| rs3760982 | G | -0.05049 | -0.04887 | -0.08038 |
| rs10941679 | G | 0.14228 | 0.16843 | 0.03244 |
| rs71338792 | AT | 0.04903 | 0.05159 | 0.03986 |
| rs28512361 | A | 0.04968 | 0.04735 | 0.08436 |
| rs1707302 | G | 0.03643 | 0.04336 | -0.00767 |
| rs6796502 | A | -0.0844 | -0.08709 | -0.08338 |
| rs6122906 | G | 0.04874 | 0.04254 | 0.06884 |
| rs72749841 | C | -0.06783 | -0.06625 | -0.01316 |
| rs35951924 | AT | -0.05293 | -0.05457 | -0.04148 |
| rs140850326 | C | -0.0324 | -0.03417 | -0.03724 |
| rs4784227 | T | 0.2044 | 0.22318 | 0.13483 |
| rs2787486 | C | -0.0762 | -0.09065 | -0.03684 |
| rs17817449 | G | -0.0537 | -0.05342 | -0.06975 |
| rs11075995 | T | -0.03376 | -0.01545 | -0.06788 |
| rs28539243 | A | 0.04943 | 0.05072 | 0.04571 |
| rs62355902 | T | 0.16344 | 0.1984 | 0.0596 |
| rs2432539 | G | -0.03342 | -0.04189 | -0.02273 |
| rs10472076 | C | 0.02513 | 0.03117 | 0.03422 |
| rs1353747 | G | -0.0413 | -0.05456 | -0.02424 |
| rs1053338 | G | 0.04514 | 0.03412 | 0.03119 |
| rs10995201 | G | -0.10601 | -0.11461 | -0.06759 |
| rs3903072 | T | -0.02988 | -0.03805 | -0.00952 |
| rs2588809 | C | -0.05622 | -0.06439 | 0.00912 |
| rs999737 | T | -0.09552 | -0.0992 | -0.07797 |
| rs75915166 | A | 0.24973 | 0.29825 | -0.01066 |
| rs6805189 | C | -0.03247 | -0.03733 | -0.00477 |
| rs6562760 | G | 0.04724 | 0.04854 | 0.08854 |
| rs6472903 | T | 0.0618 | 0.069 | 0.03811 |
| rs2943559 | G | 0.09666 | 0.09705 | 0.09485 |
| rs745570 | G | -0.02764 | -0.02056 | -0.03921 |
| rs13329835 | G | 0.06927 | 0.07685 | 0.05555 |
| rs704010 | C | -0.07148 | -0.07474 | -0.05744 |
| rs12207986 | A | 0.0336 | 0.03244 | 0.03745 |
| rs7707921 | A | 0.04021 | 0.04961 | 0.03351 |
| rs17529111 | C | 0.02194 | 0.00851 | 0.06134 |
| 4:84370124 | TA | -0.03488 | -0.03449 | -0.02632 |
| rs202049448 | C | -0.05204 | -0.04949 | -0.07232 |
| rs13066793 | G | -0.06161 | -0.07509 | -0.04115 |
| rs4496150 | A | -0.0433 | -0.03752 | -0.04449 |
| rs17426269 | A | 0.04726 | 0.06145 | 0.03551 |
| rs10022462 | T | 0.04011 | 0.04252 | 0.01201 |
| rs10474352 | T | -0.05862 | -0.06588 | -0.01866 |
| rs2290203 | A | -0.05839 | -0.06678 | -0.03869 |
| rs6964587 | T | 0.03395 | 0.03171 | 0.0156 |
| rs941764 | G | 0.03356 | 0.03318 | 0.00912 |
| rs11627032 | C | -0.03915 | -0.04122 | -0.04929 |
| rs17268829 | C | 0.04834 | 0.05807 | 0.01309 |
| rs140936696 | C | -0.04083 | -0.04507 | -0.03648 |
| rs17356907 | G | -0.0906 | -0.08751 | -0.06055 |
| rs9833888 | T | 0.05556 | 0.06695 | 0.01621 |
| rs71559437 | A | -0.06785 | -0.08153 | -0.04907 |
| rs514192 | T | -0.04424 | -0.0546 | -0.01606 |
| rs10623258 | CTT | 0.0381 | 0.03579 | 0.02967 |
| rs9790517 | T | 0.03486 | 0.05186 | -0.02314 |
| rs12546444 | T | -0.06933 | -0.07761 | -0.02324 |
| rs10759243 | A | 0.0614 | 0.07197 | 0.02285 |
| rs10816625 | G | 0.10113 | 0.11836 | 0.0716 |
| rs13294895 | T | 0.05597 | 0.07104 | -0.00678 |
| rs676256 | T | 0.08906 | 0.10944 | 0.02455 |
| rs6882649 | T | 0.02881 | 0.02863 | 0.02399 |
| rs71801447 | C | 0.0831 | 0.0845 | 0.04618 |
| rs11552449 | T | 0.0367 | 0.03509 | 0.03893 |
| rs7904519 | G | 0.03004 | 0.0201 | 0.07291 |
| rs1292011 | G | -0.08245 | -0.09905 | -0.02406 |
| rs13267382 | G | -0.03129 | -0.03549 | -0.01772 |
| rs7529522 | C | 0.06048 | 0.06673 | 0.05323 |
| rs1895062 | G | -0.06633 | -0.06572 | -0.07283 |
| rs206966 | T | 0.04464 | 0.05467 | 0.04159 |
| rs4849887 | C | 0.09619 | 0.07838 | 0.15763 |
| rs11249433 | G | 0.10649 | 0.13076 | 0.02108 |
| rs11199914 | T | -0.04115 | -0.04929 | -0.01689 |
| rs2981578 | T | -0.20635 | -0.24958 | -0.03663 |
| rs35054928 | G | -0.24233 | -0.29145 | -0.06263 |
| rs45631563 | T | -0.20712 | -0.26528 | -0.07648 |
| rs58847541 | A | 0.07327 | 0.06705 | 0.12292 |
| rs77528541 | T | -0.05627 | -0.05692 | -0.06676 |
| rs13281615 | G | 0.10035 | 0.107 | 0.06422 |
| rs11780156 | T | 0.04974 | 0.04837 | 0.04981 |
| rs10760444 | A | -0.03297 | -0.02564 | -0.04762 |
| rs11820646 | C | 0.03888 | 0.03133 | 0.06243 |
| rs6569648 | T | 0.05848 | 0.05592 | 0.06115 |
| rs4593472 | T | -0.03184 | -0.03312 | -0.03343 |
| rs6596100 | T | -0.05744 | -0.06426 | -0.0513 |
| rs8176636 | D10 | 0.03291 | 0.03445 | 0.04833 |
| rs11977670 | A | 0.06096 | 0.06162 | 0.02061 |
| rs34207738 | C | 0.05547 | 0.06046 | 0.03208 |
| rs720475 | A | -0.03749 | -0.03807 | -0.00482 |
| rs12405132 | T | -0.03075 | -0.03257 | -0.00466 |
| rs9485372 | A | -0.04584 | -0.05348 | -0.01384 |
| rs12048493 | C | 0.04183 | 0.04303 | 0.05211 |
| rs3757322 | G | 0.07948 | 0.05712 | 0.12881 |
| rs9397437 | A | 0.16059 | 0.1318 | 0.27995 |
| rs2747652 | C | 0.06131 | 0.05406 | 0.08666 |
| rs4971059 | A | 0.05186 | 0.06252 | 0.03135 |
| rs1432679 | T | -0.07649 | -0.07146 | -0.07567 |
| rs4562056 | T | 0.0481 | 0.05522 | 0.01712 |
| rs58058861 | A | 0.06186 | 0.06787 | -0.00314 |
| rs2016394 | A | -0.04629 | -0.05895 | 0.00033 |
| rs1550623 | A | 0.04915 | 0.06249 | 0.00433 |
| rs6828523 | A | -0.09718 | -0.12741 | 0.00214 |
| rs35383942 | T | 0.11704 | 0.09518 | 0.13807 |
| rs1830298 | T | -0.0561 | -0.05041 | -0.07454 |
| rs6678914 | A | 0.00317 | 0.02329 | -0.06453 |
| rs4951011 | G | 0.04219 | 0.03347 | 0.06511 |
| rs4245739 | A | -0.02282 | -0.00138 | -0.11742 |
| rs11117758 | A | -0.0548 | -0.06115 | -0.02001 |
| rs4442975 | T | -0.11867 | -0.14289 | -0.05665 |
| rs34005590 | A | -0.19504 | -0.2274 | -0.02342 |
| rs16857609 | T | 0.06176 | 0.06638 | 0.06376 |
| rs12479355 | G | -0.03876 | -0.04335 | -0.00213 |
| rs72755295 | G | 0.13592 | 0.1523 | 0.08797 |

Table S3. The association between hyperthyroidism and major breast cancer risk predictors among the KARMA cohort, by type of hyperthyroidism (N=67,518)

|  | Graves’ disease | |  | Toxic nodular goiter | |  | Other types | |
| --- | --- | --- | --- | --- | --- | --- | --- | --- |
| Variable names | No. | OR (95%CI) |  | No. | OR (95%CI) |  | No. | OR (95%CI) |
| Benign breast disease |  |  |  |  |  |  |  |  |
| No | 335 | 1.00 (REF) |  | 104 | 1.00 (REF) |  | 139 | 1.00 (REF) |
| Yes | 96 | 0.99 (0.79-1.25) |  | 43 | 1.28 (0.90-1.84) |  | 36 | 0.88 (0.61-1.28) |
| Number of births |  |  |  |  |  |  |  |  |
| 0 | 58 | 1.00 (REF) |  | 19 | 1.00 (REF) |  | 25 | 1.00 (REF) |
| 1 | 67 | 1.02 (0.72-1.45) |  | 21 | 0.91 (0.49-1.70) |  | 36 | 1.25 (0.75-2.08) |
| 2 | 225 | 1.06 (0.79-1.42) |  | 74 | 1.03 (0.62-1.71) |  | 87 | 0.95 (0.61-1.49) |
| >2 | 93 | 0.84 (0.60-1.17) |  | 35 | 0.89 (0.51-1.57) |  | 32 | 0.66 (0.39-1.12) |
| Age at first birth* (years) |  |  |  |  |  |  |  |  |
| <25 | 148 | 1.00 (REF) |  | 64 | 1.00 (REF) |  | 54 | 1.00 (REF) |
| 25-29 | 113 | **0.74 (0.57-0.95)** |  | 39 | 0.67 (0.44-1.00) |  | 50 | 0.91 (0.61-1.35) |
| >=30 | 124 | 0.93 (0.71-1.22) |  | 27 | 0.65 (0.39-1.06) |  | 51 | 1.01 (0.65-1.55) |
| Breastfeeding* duration (months) |  |  |  |  |  |  |  |  |
| <1 | 44 | 1.00 (REF) |  | 18 | 1.00 (REF) |  | 14 | 1.00 (REF) |
| 1-6 | 148 | 0.81 (0.58-1.14) |  | 53 | 0.73 (0.43-1.26) |  | 69 | 1.26 (0.71-2.26) |
| >6 | 190 | **0.69 (0.49-0.98)** |  | 59 | 0.78 (0.45-1.36) |  | 71 | 0.88 (0.48-1.60) |
| BMI (kg/cm^2^) category |  |  |  |  |  |  |  |  |
| <18.5 | 5 | 1.15 (0.47-2.81) |  | 3 | 2.26 (0.71-7.24) |  | 1 | 0.57 (0.08-4.12) |
| 18.5-25 | 235 | 1.00 (REF) |  | 68 | 1.00 (REF) |  | 95 | 1.00 (REF) |
| 25-30 | 140 | 1.03 (0.83-1.27) |  | 49 | 1.14 (0.79-1.65) |  | 54 | 0.97 (0.70-1.36) |
| >30 | 61 | 1.07 (0.81-1.43) |  | 29 | **1.72 (1.11-2.67)** |  | 30 | 1.30 (0.86-1.97) |
| Family history of breast cancer |  |  |  |  |  |  |  |  |
| No | 363 | 1.00 (REF) |  | 127 | 1.00 (REF) |  | 147 | 1.00 (REF) |
| Yes | 58 | 1.06 (0.81-1.41) |  | 18 | 0.88 (0.54-1.45) |  | 20 | 0.91 (0.57-1.46) |
| Oral contraceptive use |  |  |  |  |  |  |  |  |
| No | 92 | 1.00 (REF) |  | 35 | 1.00 (REF) |  | 29 | 1.00 (REF) |
| Yes | 351 | 0.94 (0.74-1.19) |  | 114 | 0.99 (0.67-1.46) |  | 150 | 1.29 (0.86-1.93) |
| Hormone replacement therapy use |  |  |  |  |  |  |  |  |
| No | 320 | 1.00 (REF) |  | 88 | 1.00 (REF) |  | 122 | 1.00 (REF) |
| Yes | 121 | 0.95 (0.75-1.19) |  | 58 | 1.09 (0.77-1.56) |  | 56 | 1.12 (0.79-1.58) |
| Age at menarche (years) |  |  |  |  |  |  |  |  |
| <12 | 168 | 1.00 (REF) |  | 52 | 1.00 (REF) |  | 68 | 1.00 (REF) |
| 13-16 | 247 | 0.85 (0.70-1.04) |  | 90 | 0.94 (0.67-1.33) |  | 99 | 0.87 (0.64-1.19) |
| >16 | 21 | 0.88 (0.56-1.40) |  | 4 | 0.49 (0.18-1.37) |  | 11 | 1.22 (0.64-2.32) |
| Menopausal status |  |  |  |  |  |  |  |  |
| Pre-menopausal | 192 | 1.00 (REF) |  | 32 | 1.00 (REF) |  | 72 | 1.00 (REF) |
| Peri-menopausal | 28 | 1.13 (0.73-1.73) |  | 5 | 0.85 (0.32-2.28) |  | 17 | 1.75 (0.98-3.12) |
| Post-menopausal | 260 | 1.21 (0.87-1.68) |  | 125 | 1.73 (0.94-3.18) |  | 109 | 1.31 (0.78-2.19) |
| History of irregular menstrual periods |  |  |  |  |  |  |  |  |
| No | 374 | 1.00 (REF) |  | 138 | 1.00 (REF) |  | 159 | 1.00 (REF) |
| Yes | 60 | 1.21 (0.92-1.59) |  | 14 | 0.87 (0.50-1.52) |  | 22 | 1.03 (0.66-1.62) |

OR: odds radio; BMI: body mass index. ORs were calculated in a multivariate model including all risk predictors simultaneously.

*Analyses for age at first birth and breastfeeding duration were limited to parous women.

Table S4. The association between hyperthyroidism and mammographic density among KARMA women without cancer, by type of hyperthyroidism (n=51,928)

|  |  | Graves’ disease | |  | Toxic nodular goiter | |  | Other types | |
| --- | --- | --- | --- | --- | --- | --- | --- | --- | --- |
|  | N | No. | OR (95% CI) |  | No. | OR (95% CI) |  | No. | OR (95% CI) |
| **Mammographic density** |  |  |  |  |  |  |  |  |  |
| **Q 1** | 12797 | 129 | 1.00 (REF) |  | 54 | 1.00 (REF) |  | 61 | 1.00 (REF) |
| **Q 2** | 12801 | 130 | 1.08 (0.84-1.40) |  | 43 | 1.07 (0.71-1.62) |  | 69 | 1.22 (0.85-1.74) |
| **Q 3** | 12807 | 121 | 1.07 (0.81-1.41) |  | 47 | **1.71 (1.10-2.66)** |  | 60 | 1.10 (0.74-1.64) |
| **Q 4** | 12825 | 126 | 1.15 (0.85-1.57) |  | 31 | **1.79 (1.03-3.11)** |  | 51 | 1.00 (0.64-1.58) |
| **P for Square root continuous** |  |  | 0.30 |  |  | **0.01** |  |  | 0.57 |

The Model is adjusted for age at mammogram, BMI, age at menarche, number of births, family history of breast cancer, HRT use, oral contraceptive use, and benign breast disease.

Fig. S1. Sample attrition for analyses using the KARMA cohort

KARMA cohort

(N=70,877)

KARMA cohort for breast cancer risk analysis 2002-2017 (N=69,598; corresponding to results in Table 1)

Exclusion criteria:

- Women diagnosed with breast cancer before they participate in KARMA (n=2,080)

KARMA women without breast cancer when entering the KARMA study (N=67,518; corresponding to results in Table 2)

For mammographic density analysis:

Exclusion criteria:

- No mammographic data (N=1,110)
- Breast operation or missing information (N=11,861)
- Other cancer (N=2,325)
- No BMI data (N=294)

For genetic analysis:

Exclusion criteria:

- No genotyped data (N=55,527)

KARMA women without breast cancer who had genotyped data available (N= 11,991; corresponding to results in Table 4)

KARMA women without cancer who had mammographic density data available (N= 51,928; corresponding to results in Table 3)
